# miR-19b enhances proliferation and apoptosis resistance via the EGFR signaling pathway by targeting PP2A and BIM in non-small cell lung cancer

**DOI:** 10.1186/s12943-018-0781-5

**Published:** 2018-02-19

**Authors:** Ulrich Baumgartner, Fabienne Berger, Ali Hashemi Gheinani, Sabrina Sofia Burgener, Katia Monastyrskaya, Erik Vassella

**Affiliations:** 1 Institute of Pathology, University of Bern, Bern, Switzerland; 2 Graduate School for Cellular and Biomedical Sciences, University of Bern, Bern, Switzerland; 3 Urology Research Laboratory, University Hospital, Bern, Switzerland; 4 Institute for Virology and Immunology, Vetsuisse Faculty, University of Bern, Mittelhäusern, Bern, Switzerland; 5 Institut für Pathologie, University of Bern, Murtenstrasse 31, CH-3008 Bern, Switzerland

**Keywords:** Apoptosis, Proliferation, Epidermal growth factor receptor, microRNA, Non-small cell lung cancer, Serine-threonine phosphatase

## Abstract

**Background:**

Epidermal growth factor receptor (*EGFR*) mutations enable constitutive active downstream signaling of PI3K/AKT, KRAS/ERK and JAK/STAT pathways, and promote tumor progression by inducing uncontrolled proliferation, evasion of apoptosis and migration of non-small cell lung cancer (NSCLC). In addition, such *EGFR* mutations increase the susceptibility of patients with NSCLC to tyrosine kinase inhibitor (TKI) therapy, but treated patients will invariably relapse with resistant disease. A global understanding of underlying molecular mechanisms of EGFR signaling may improve the management of NSCLC patients.

**Methods:**

microarray analysis was performed to identify PI3K/AKT-regulated miRNAs. Phosphoproteomic analysis and cell based assays were performed using NSCLC cell lines lentivirally transduced with anti-miR or miR overexpressing constructs.

**Results:**

Here, we show that 17 miRNAs including members of the miR-17~ 92 cluster are dysregulated following PI3K/AKT inhibition of EGFR mutant NSCLC cells. Bioinformatics analysis revealed that dysregulated miRNAs act in a concerted manner to enhance the activity of the EGFR signaling pathway. These findings were closely mirrored by attenuation of miR-17~ 92 family member miR-19b in NSCLC cell lines which resulted in reduced phosphorylation of ERK, AKT and STAT and effector proteins in *EGFR* mutant NSCLC cells. Consistent with this finding, cell cycle progression, clonogenic growth and migration were reduced and apoptosis was enhanced. Co-treatment of NSCLC cells with the tyrosine kinase inhibitor (TKI) gefitinib and anti-miR-19b construct reduced migration and clonogenic growth in a synergistic manner suggesting that EGFR and miR-19b act together to control oncogenic processes. Serine/threonine phosphatase PP2A subunit *PPP2R5E* and *BCL2L11* encoding BIM were identified as major targets of miR-19b by target validation assays. Consistent with this finding, PP2A activity was strongly enhanced in NSCLC transduced with anti-miR-19b construct, but not in cells co-transduced with anti-miR-19b and *shPPP2R5E*, suggesting that PPP2R5E is a major constituent of the PP2A complex. Accordingly, enhanced proliferation by miR-19b was due to targeting *PPP2R5E*. In contrast, apoptosis resistance was mainly due to targeting *BCL2L11*.

**Conclusion:**

Our results provide insight into the importance of targeting *PPP2R5E* and *BCL2L11* by miR-19b in oncogenic processes of NSCLC. Attenuation of miR-19b expression could potentially be exploited in adjuvant therapy of *EGFR* mutant NSCLC.

**Electronic supplementary material:**

The online version of this article (10.1186/s12943-018-0781-5) contains supplementary material, which is available to authorized users.

## Background

Lung cancer is the leading cause of cancer-related death worldwide with a mean 5-year survival rate of less than 15% [1]. Platinum-based therapy is the standard of care for patients with metastatic non-small cell lung cancer (NSCLC), the most common subtype of lung cancer [2]. The introduction of targeted therapy using tyrosine kinase inhibitors (TKI) increased overall survival of patients with metastatic NSCLC harboring activating mutations in the epidermal growth factor receptor (EGFR) compared with standard cytotoxic therapy [3]. Nevertheless, 25% of these patients respond poorly to the therapy and virtually all patients eventually relapse owing to acquisition of secondary EGFR mutations or reactivation of signaling pathways downstream of EGFR [4, 5]. Thus, despite promising initial clinical responses in some patients, the 5-year survival rate of patients treated with TKI remains relatively low [6]. A deeper understanding of underlying molecular processes of EGFR signaling may provide insights into improving the management of EGFR-mutant lung cancer patients.

The EGFR signaling pathway is among the most important drivers of lung tumorigenesis: mutations in *EGFR* (10–15%) or mutations or translocations of downstream effectors including *KRAS* (25–40%) and *ALK* (5–7%) are frequently found in Caucasian NSCLC patients [7]. This results in overactivation of effector pathways including the RAS/ERK, JAK/STAT AKT/mTOR pathway, and enhancement of five of six hallmarks of cancer including evasion of apoptosis, sustained angiogenesis, resistance to antigrowth signals, invasion and metastasis and self-sufficiency in growth signals [4].

The activity of kinases in the EGFR signaling pathway is controlled by phosphatases, which remove the phosphate groups within minutes after phosphorylation [8]. Thus, kinases and phosphatases are equally important in modulating the activity of signaling pathways, but the role of phosphatases is far less understood. Serine/threonine phosphatase PP2A is a heterotrimeric protein composed of a structural subunit A, a catalytic subunit C and a regulatory subunit B. Members of the regulatory B subunit exhibit tissue-specific expression profiles, and are implicated in diverse cellular functions by recruiting PP2A to specific substrates [9]. PP2A is a critical regulator of ERK and AKT, and controls downstream effectors of EGFR including NF-κB, TP53 and Bcl2 [9–11]. The importance of PP2A in EGFR signaling is also illustrated by the finding that administering SMAPs, small molecule activators of PP2A, results in substantial inhibition of KRAS-driven tumor growth [12]. Conversely, procadherin 7, an endogenous inhibitor of PP2A, which acts through SET, potentiates ERK signaling through EGFR and KRAS, and promotes transformation of KRAS transduced bronchial epithelial cells [13]. Consistent with these findings, PP2A is repressed in NSCLC by inactivating mutations, overexpression of PP2A inhibitory proteins or post-translational modifications [14], but in most cases the underlying molecular mechanisms are unknown.

MicroRNAs (miRNAs), short regulatory RNA sequences, which control gene expression at the post-transcriptional level, are critical regulators of signaling pathways. They act as signal amplifiers or attenuators and promote the cross-talk between signaling pathways [15]. In a previous study, we showed that miR-29b is a mediator of NF-κB signaling in KRAS-transduced NSCLC [16]. In this study, we define miR-19b as a mediator of the PI3K/AKT signaling pathway. miR-19b is the major oncogenic miRNA of the miR-17-92 cluster, and plays a central role in tumorigenesis of B-cell lymphomas [17–19]. miR-19b is also an oncogenic miRNA in NSCLC, and is implicated in proliferation [20], attenuation of apoptosis and migration [21]. Upregulation of miR-19b and its paralogue miR-19a in the tumor tissue as well as in the serum is associated with poor prognosis of patients with NSCLC [22–24]. Here we report that miR-19b potentiates EGFR signaling by targeting PP2A B subunit PPP2R5E and confers apoptosis resistance by targeting BCL2L11 encoding the BH3 domain-containing protein BIM. Our results provide insight into oncogenic processes of miR-19b in NSCLC cells.

## Methods

### Cell lines and drug treatment

EGFR mutant NSCLC cell lines PC9 and PC9ER (kindly provided by PD Dr. A. Arcaro, Department of Clinical Research, University of Bern, Bern, Switzerland), HCC4011 (kindly provided by Prof. M.D. A. F. Gazdar and Prof. M.D. J. Minna, University of Texas Southwestern Medical Center, Dallas, TX, USA) and HCC827 (American Type Culture Collection, Manassas, VA, USA) were used in this study. All cell lines were cultured in complete Roswell Park Memorial Institute medium (cRPMI) (Sigma-Aldrich, Buchs, Switzerland), supplemented with 4 mmol/l L-alanyl-L-glutamine (Bioswisstec AG, Schaffhausen, Switzerland) 1% penicillin/streptomycin and 10% fetal bovine serum (Sigma-Aldrich) at 37 °C and 5–10% CO_2_. Cell lines were authenticated by STR profiling (Microsynth, Balgach, Switzerland) in March 2016.

EGFR inhibitors Gefitinib (Selleckchem, Munich, Germany) and Afatinib (Selleckchem), PI3K-inhibitor LY294002 (Selleckchem), and MEK-inhibitor U0126 (Selleckchem) were used at concentrations indicated in the text.

### Constructs

Luciferase reporter constructs were obtained by cloning double-stranded oligonucleotides encompassing the wild type or mutated miR-19b target sites from PPP2R5E or BCL2L11, respectively, into the *Xba*I and *Xho*I sites of pmiRGLO Dual-Luciferase miRNA target expression vector (Promega, Dübendorf, Switzerland). Lentiviral expression vector hsa-miR-19b-NW was obtained by cloning a PCR product encompassing the pri-miRNA sequence of miR-19b into the *Not*I and *Eco*RI sites of PMIRH125b-1PA-1. Oligonucleotides used for cloning are indicated in Additional file 1: Table S1. Antisense hsa-miR-19b and antisense scrambled control (System Biosciences, San Francisco, CA) were used for attenuation of miR-19b-3p levels. Gene knockdown experiments were performed using shPPP2R5E, shBCL2L11 and shc002 constructs (Sigma-Alderich, Buchs, Switzerland).

### Transfections and luciferase assays

NSCLC cells were transfected with 100 ng pmiRGLO vector using transfection reagent HiPerFect (Qiagen, Hombrechtikon, Switzerland) according to the fast-forward protocol provided by the supplier. Luciferase reporter assays were performed 48 h post transfection [25].

### Lentiviral transduction and cell-based assays

Lentiviral production was carried out as described [26]. Transduction efficiency was assessed for GFP expression 3 days post transduction by FACS. Transduced cells were sorted by FACS or selected with 0.5 μg/mL puromycin (Sigma-Aldrich).

Apoptosis was induced by treating cells with 10 ng/ml TNFα (PeproTech, Rocky Hill, NJ, USA) in combination with 0.5 μg/mL actinomycin D (Sigma-Aldrich) for 6 h. Apoptosis and viability were assessed using the ApoTox-Glo Triplex assay (Promega) as described [25]. Alternatively, apoptosis was assessed using the pacific blue annexin V apopotosis detection kit with PI (LucernaChem). Annexin V/propidim iodide-positive cells were analyzed using a LSR II Flow Cytometer (Becton Dickinson) and FlowJo software version9.8.2 (Tree Star).

Anchorage-dependent clonogenic assay was performed in six-well plates seeded with transduced cells and cultured for 10 days in cRPMI. Colonies were fixed with methanol and stained with 0.5% crystal violet solution (Sigma-Aldrich) for 30 min, washed with deionized water and lysed in 1 mL 1% (*W*/*V*) SDS. Clonogenic growth was assessed by measuring the absorption of the lysate at 505 nm using an Infinite 200 PRO plate reader (TECAN, Männedorf, Switzerland). At least three independent experiments were carried out for each experiment.

Cell proliferation was assessed by 5-bromo-2-deoxyuridine (BrdU) incorporation assay according to the manufacturer’s instructions (Roche Diagnostics). Four thousand cells were plated per well of a 96-well plate. BrdU incorporation was performed one day post-seeding for 5 h. At least three independent experiments were carried out for each experiment.

Wound healing assay was performed as described [27]. Sixty thousand cells were allowed to adhere for 4–6 h in a 100 μL drop of cRPMI placed in the middle of a 6-well culture dish. The monolayer was artificially injured by scratching across the plate with a 200 μL pipette tip. Wells were washed twice with cRPMI to remove detached cells and wound healing was monitored over a period of 24 h using the imaging system Cell-IQ (Canibra, Bramsche, Germany) and the CellActivision software version R1.03.01 (Yokogawa Electric Corporation, Republic of Korea).

### Phosphatase activity assay

Cell extracts were prepared as described [28]. Following centrifugation for 10 min at 12000 g, the soluble fraction was passed through a NucAwayTM Spin column (Fisher Scientific, Reinach, Switzerland) equilibrated with storage buffer and the protein concentration in the eluate was determined using the Qubit protein assay (ThermoFisher). 15 ng of the eluate was analyzed using the Ser/Thr phosphatase assay (Promega) according to manufacturer’s instructions. Cell lysates were pre-incubated at 37 °C for 10 min and the reaction was continued in the presence of PP2A substrate for 2 h. Phosphatase activity was also assessed in the presence of 25 μM PP2A inhibitor LB-100 (Selleckchem). The reaction was stopped by the addition of molybdate dye and released P_i_ was quantified by absorption spectroscopy at 600 nm. Phosphatase activity in the presence of P_i_ depleted H_2_O was used as a blank. The assay was linear for the indicated incubation period and the amount of protein extract.

### Phospho-kinase array and western blot analysis

Phospho-kinase array analysis was performed using 800 μg total protein according to manufacturer’s instructions (R&D Systems, Zug, Switzerland). Briefly, cell lysates were mixed with biotinylated detection antibodies and phospho-proteins were captured using antibodies spotted in duplicate on nitrocellulose membranes and quantified by chemoluminescence. Following background subtraction, the average signal intensity of pair of duplicate spots was normalized to the overall signal intensity.

For Western blot analysis 20 μg total protein was loaded per lane on a 4–20% Mini-PROTEAN TGX Gel (Bio-Rad Laboratories AG, Reinach, Switzerland). Separated proteins were transferred to PVDF membranes using the transfer turbo system (Bio-Rad). Monoclonal antibodies used in this study were directed against AKT (40D4, 1:1000, Cell Signaling Technologies), phospho-AKT (D7F10, Ser473, 1:1000, CST), CCND1 (SP4, 1:100, Cell Marque), ERK1/2 (L34F12, 1:2000, CST), phospho-ERK1/2 (D13.14.4E, Thr202/Tyr204, 1:2000, CST), GSK3β (3D10, 1:1000, CST), phospho-GSK3β (D85E12, Ser9, 1:1000, CST), PPP2R5E (5A5-1F3, 1:1000, Millipore), BIM (C34C5, 1:1000, CST), PTEN (138G6, 1:1000, CST), S6 Ribosomal protein (54D2, 1:1000, CST), phospho-S6 Ribosomal protein (D57.2.2E, Ser235/236, 1:1000, CST), STAT3 (124H6, 1:1000, CST), phospho-STAT3 (D3A7, Tyr705, 1:1000, CST), α-tubulin (clone DM1A, 1:1000, CST), GAPDH (clone D16H11, 1:1000, CST). Secondary polyclonal- donkey anti-rabbit-HRP and donkey anti-mouse-HRP (Jackson Immuno Research, Suffolk, UK) were used at 1:5000. Protein levels were normalized to α-tubulin. Visualization and quantification of protein bands were performed using a luminescent image analyzer LAS-4000 (Fujifilm, Dielsdorf, Switzerland) and Multi Gauge software (Fujifilm v.3.0).

### RNA isolation and real-time PCR

RNA extraction and real-time PCR were performed as described [29]. miRNA levels were analyzed using TaqMan Assay (Applied Biosystems), and mRNA levels were analyzed using QuantiTec Primers (Qiagen). miRNA and mRNA levels were normalized to the levels obtained for RNU48 and GAPDH, respectively. Changes in expression were calculated using the ΔΔCT method.

### High-throughput miRNA NanoString profiling

One hundred and fifty ng total RNA was analyzed using the nCounter Human miRNA Expression Assay Kit H_miRNA_V3 (NanoString, Seattle, WA, USA) according to manufacturer’s instruction. Each sample was scanned for 555 fields of view (FOV) using the nCounter Digital Analyzer. nCounter data imaging QC metrics revealed no significant discrepancy between the FOVs attempted, and the FOVs counted. The binding density for the samples ranged between 0.08 and 0.21 within the recommended range.

### Statistical and bioinformatics analysis

#### NanoString normalization

Positive control correction was used to confirm ligation of the miRNAs to the tags. The positive correction was performed by

$$ c\times \left(\frac{m}{s}\right) $$

In this equation c is count for a microRNA in a given sample, m is the mean of the sum of the positive controls across all samples, and s is the sum of all of the positive controls for that given sample. We modified NanoStriDE web application and implemented DESeq ANODEV (uses DESeq’s built in normalization methods) in an R script. Negative control (unique probes for which no target sequence is present in the human transcriptome) subtraction and normalization of positive control corrected data were performed using “NanoStringNorm” and “NanoStringDiff” R packages (available in CRAN). We used the mean of the negative controls summed with 2 standard deviations of the negative controls. mRNA sequences (ACTB, B2M, GAPDH, RPL19 and RPLP0) were used to confirm successful hybridization and to normalize variations in sample input.

#### Differential expression profiling of normalized microRNAs

The normalized count data were modelled over-dispersed Poisson data using a negative binomial model in the EdgeR Bioconductor package.

#### Hierarchical clustering and heatmap

Hierarchical clustering and the associated heatmap for miRNA profiling data was generated with the function heatmap2 in the R package gplots or GENE-E R package [30]. We used pairwise correlation matrix between items based on Pearson correlation method. The correlation matrix was converted as a distance matrix. Finally, clustering was calculated on the resulting distance matrix. We used average linkage method to calculate the distance matrix.

#### Volcano graph

miRNA content in DMSO treated cells were compared to PI3K inhibitor treated cells.

-log10 adjusted *p* value was plot against log2 fold change of corresponding samples using a custom R function.

#### Prediction of altered canonical pathways based on differentially expressed microRNAs

The prediction of targets of differentially regulated microRNAs was done by TargetScan and the experimentally observed relationships was collected from TarBase. The significance values for the canonical pathways was calculated by Fisher’s exact test right-tailed. The significance indicates the probability of association of microRNA targets from our dataset with the canonical pathway by random chance alone. For Nanostring dataset, the intensity of alteration of mRNAs of each canonical pathway was calculated based on reverse regulation of microRNA fold changes. An ‘enrichment’ score [Fisher’s exact test (FET) *P*-value] that measures overlap of observed and predicted regulated gene sets was calululated.

#### Pathway analysis based on the dataset from the phosphatase array

In order to identify upstream regulators and causal network master regulators that can potentially create the changes in phosphorylation levels of the proteins in our phosphoproteomics dataset, Phosphorylation Core Analysis tool in IPA was used to predict the affected canonical pathways [30].

#### Word cloud

To visualize gene enrichment data from a pathway analysis dataset, a cloud was created using Wordle.net and Word cloud R package. The font size of a gene (tag) is determined by its incidence in the pathway analysis data set.

#### Prediction of biological function of canonical pathways

We used “BioFun” R Package (available upon request) tool that examines involvement of each IPA canonical pathway in the Biological Function Classification Database of IPA known as “Ingenuity canonical pathway” and counts the number of pathways involved in a specific biological function. The results are illustrated as radar graphs.

#### Statistical differences

Statistical differences were calculated using the unpaired two-tailed Student’s t-test in GraphPad Prism software (v.7.0a). Statistical significance was achieved at a probability of *, *P* < 0.05; **, *P* < 0.01; ***, *P* < 0.001; ****, *P* < 0.0001; ns, not significant.

## Results

### Expression profiling of PI3K/AKT effector miRNAs

**Fig. 1:**
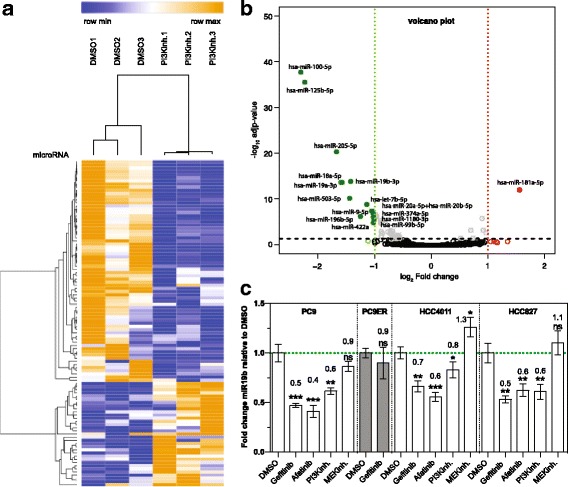
miR-19b is regulated by the PI3K/AKT branch of the EGFR signaling pathway. **a** Heat map clustering of miRNAs that are dysregulated by the PI3K inhibitor LY294002. PC9 cells were treated with 20 μM LY294002 or DMSO control for 72 h and expression of 800 miRNAs was analyzed by NanoString using the nCounter Human miRNA Expression Assay Kit (*n* = 3). **b** Volcano plot of dysregulated miRNAs described in (A) using a cutoff ±0.4 log2 fold change (FC) (n = 3). **c** miR-19b expression level by real-time qPCR relative to RNU48 in *EGFR* mutant NSCLC cells treated with 1.25 μM gefitinib, 1.25 uM afatinib, 20 μM LY294002 and 10 μM U0126 for 72 h. Results are presented as mean ± SD (*n* = 3). ns, not significant. *, *p* < 0.05; **, *p* < 0.01; ***, *p* < 0.001

To identify effector miRNAs of the PI3K/AKT pathway, the NSCLC cell line PC9 containing a constitutive active EGFR mutation was treated with the PI3K inhibitor LY294002 and changes in global miRNA expression was assessed using the NanoString technology. Thirty-three miRNAs were upregulated and 71 miRNAs were downregulated by LY294002. Hierarchical clustering and heatmap analysis revealed clear distinction of both experimental groups (Fig. 1a). A volcano plot was constructed to display fold change and *p*-value, which allowed the identification of 16 miRNAs that were downregulated and 1 miRNA that was upregulated using a cutoff ±0.4 log2 FC (Fig. 1b, and Table 1). miR-100-5p, miR-125-5p, miR-205-5p, miR-19b-3p, miR-7b-5p, miR-9-5p, miR-20a/b-5p and miR-374a-5p, which were downregulated in previous studies using EGFR knockdown cell lines [31–33], were also significantly downregulated by LY294002, but their role in PI3K/AKT signalling has not yet been addressed.

**Table 1:** Top miRNAs regulated by the PI3K inhibitor LY294002

Rank	miRNAs	Fold change (log2)^1^	adjusted *p*-value
1	hsa-miR-100-5p	−2.30	2.15E-38
2	hsa-miR-125b-5p	−2.23	3.40E-36
3	hsa-miR-205-5p	−1.67	5.63E-21
4	hsa-miR-19a-3p^2^	−1.59	2.28E-14
5	hsa-miR-18a-5p^2^	−1.57	2.28E-14
6	hsa-miR-181a-5p	1.56	8.78E-13
7	hsa-miR-503-5p	−1.44	7.17E-11
8	hsa-miR-19b-3p^2^	−1.43	1.50E-14
9	hsa-miR-196b-5p	−1.25	6.66E-07
10	hsa-let-7b-5p	−1.14	1.73E-09
11	hsa-miR-372-3p	−1.12	1.25E-01
12	hsa-miR-9-5p	−1.05	3.65E-08
13	hsa-miR-20a-5p + hsa-miR-20b-5p^2^	−1.04	3.65E-08
14	hsa-miR-422a	−1.02	1.38E-05
15	hsa-miR-1180-3p	−1.02	6.77E-07
16	hsa-miR-374a-5p	−1.02	1.48E-07
17	hsa-miR-99b-5p	−1.01	3.42E-06

^1^Fold change relative to DMSO treated cells. Cut off was set to ±0.4 log2FC (n = 3)

^2^OncomiR-1 cluster

Interestingly, IPA revealed that there is a significant bias for LY294002-regulated miRNAs towards the EGFR signaling pathway (Additional file 2: Figure S1a). The pathways were built in silico using highly predicated and experimentally validated targets of 17 top regulated and significant miRNAs shown in Table 1. Notably, targets of these miRNAs might be involved in glioblastoma signaling (PI3K signaling pathway), STAT3 Pathway, TGF-β, ERK5, Rho Family GTPases, PTEN, ERK/MAPK, and EGF pathways. MAPK1, PIK3 subunits PIK3R3, PIK3R1, PIK3CA and PK3CB, ATM, KRAS, NRAS and FGR were involved in 70% of the regulated pathways (Additional file 2: Figure S1b). Pathway analysis information was further interrogated using the IPA Biological Function Classification Database, which allows for the identification of biological functions affected by specific miRNAs [30]. Cellular immune response, cellular growth, proliferation and development are most likely processes affected by LY294002-regulated miRNAs (Additional file 2: Figure S1c). Thus, we concluded that miRNAs that are effectors of the EGFR signaling pathway are also implicated in modulating cellular processes elicited by EGFR signaling.

Among the miRNAs that are dysregulated by LY294002, family members of the miR-17~ 92 cluster, including miR-19a, miR-18a, miR-19b, miR20a and miR-20b, seemed to be most prominent. Collective read number of the members of this miRNA cluster was 5282, making it the most abundant cluster among LY294002-regulated miRNA species (top 5% expressed miRNA). IPA conducted using the predicted targets of family members of the miR-17~ 92 cluster closely mirrored the findings obtained with LY294002-regulated miRNAs (data not shown). This is in line with previous observations that members of this miRNA cluster are among the most potent oncogenic miRNAs [18, 19].

We focused on miR-19b in subsequent experiments for the following reason: miR-19b is (i) the most important oncogenic miRNA of the miR-17~ 92 cluster [17–19], is (ii) associated with NSCLC aggressiveness [34], and is (iii) upregulated during transformation and progression of NSCLC [20]. miR-19b and its paralog miR-19a are strongly regulated by LY294002 (Table 1). Pharmacologic inhibitors of EGFR (gefitinib and afatinib) resulted in 1.4–2.5-times lower miR-19b levels in 3 independent NSCLC cell lines harboring constitutive active EGFR mutations (Fig. 1c), but miR-19b levels were not affected by gefitinib in gefitinib-resistant PC9-ER cells, confirming the specificity of the TKI. Likewise, the PI3K inhibitor LY294002 resulted in 1.3–1.7-times lower miR-19b levels consistent with the results from the Nanostring analysis. In contrast, the MEK inhibitor U0126 had no effect on the miR-19b expression level (Fig. 1c), while it significantly reduced the level of KRAS-induced miR-29b [16] under the same conditions (data not shown). None of the TKIs resulted in apparent cell death under these conditions (Additional file 3: Figure S2). In conclusion, miR-19b is regulated by the PI3/AKT branch of the EGFR signaling pathway. The promoter region of the miR-17~ 92 cluster contains binding sites for multiple transcription factors including myc, E2F, SP1 and NFY [35], but it remains to be shown if PI3/AKT affects miR-17~ 92 expression through either of these transcription factors.

### miR-19b affects phosphorylation of kinases of the EGFR signaling pathway

**Fig. 2:**
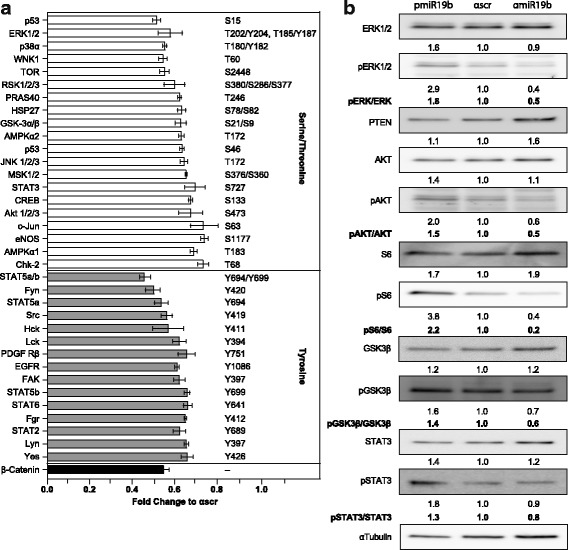
Changes in phosphorylation elicited by miR-19b. **a** Phosphoprotein array of anti-miR19b–transduced PC9 cells relative to control (*n* = 4). Signal intensity of pairs of duplicate spots is indicated. Only proteins that give rise to a signal intensity at least two fold above the background and a fold change ≤ 0.8 relative to control are indicated. **b** Western blot analysis of pre-miR-19b and anti-miR-19b-transduced cells. Protein levels were normalized to α-tubulin and presented relative to the control. Numbers below the immunoblots indicate relative expression values. Signal ratio of phospho-specific antibody and pan-specific antibody for respective proteins are indicated in bold

To assess whether miR-19b modulates the activity of the EGFR signaling pathway, phosphoproteomic analysis was performed using a phosphokinase antibody array (R&D Systems). To this end, PC9 cells were transduced with a lentivirus expressing the anti-miR-19b construct, which significantly lowered the miR-19b levels compared to the control (Additional file 4: Figure S3). Serine/threonine phosphoproteins including kinases from the major branches of the EGFR pathway such as ERK1/2, p38a, JNK1/2/3, AKT1/2/3 and STAT3 as well as downstream effectors such as p53, mTOR, S6 kinase, GSK-3a/b, c-Jun and Chk-2 were strongly reduced in the miR-19b knockdown cells (Fig. 2a). Tyrosine kinases such as EGFR were also affected.

Western blot analysis confirmed our findings of the phosphokinase antibody array (Fig. 2b). Overexpression of miR-19b resulted in enhanced phospho-ERK1/2, phospho-AKT and phospho-STAT protein levels. Consistent with these findings, phosphorylation of effectors of the AKT pathway such as pS6 and pGSK3β were significantly enhanced. Conversely, attenuation of miR-19b expression resulted in reduced phosphorylation of above mentioned proteins. Under the same experimental condition, PTEN, a known target of miR-19b, was significantly enhanced in miR-19b-attenuated cells, but overexpression of miR-19b did not significantly affected PTEN protein levels.

Activity profile of 45 phosphoproteins (shown in Fig. 2a) was submitted for pathway analysis based on phosphorylation profile of IPA platform (Additional file 5: Figure S4a). Upstream analysis of these pathways indicates EGF as the main upstream element (Additional file 5: Figure S4b). In conclusion, down-regulation of miR-19b inhibits all major branches of the EGFR signaling pathway. Interestingly, analysis of regulated pathways revealed an almost complete overlap of biological functions deduced from the phosphoprotein array in miR-19b-attenuated cells and biological functions built on targets of LY294002-regulated miRNAs (Additional file 2: Figure S1c and Additional file 5: Figure S4c), indicating that PIK3-regulated miRNAs share similar functions.

### PP2A subunit *PPP2R5E* and *BCL2L11* are major targets of miR-19b

**Fig. 3:**
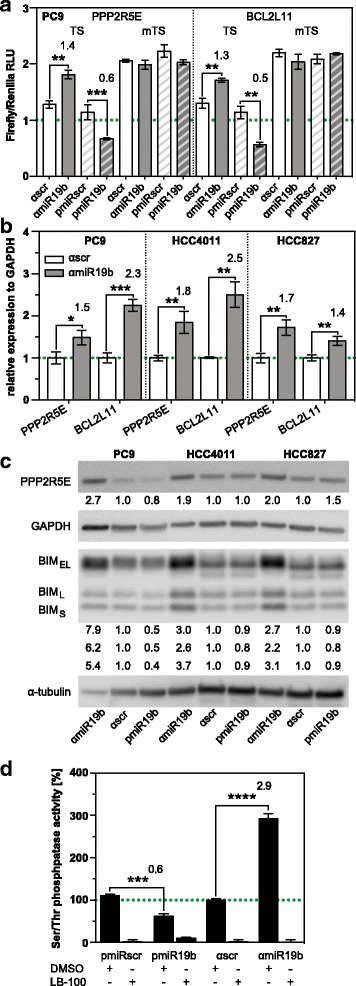
*PPP2R5E* and *BCL2L11* are direct targets of miR-19b. **a** Luciferase reporter assay of PC9 cells transduced with pre-miR19b (pmiR19b), pre-control (pmiRscr), anti-miR19b (αmiR19b) and anti-control (αscr) and transfected with constructs containing the wild-type (TS) or mutated (mTS) miR-19b target site from *PPP2R5E* or *BCL2L11*, respectively. Luciferase activity is presented as mean ± SD relative to Renilla activity (n = 3). **b** *PPP2R5E* and *BCL2L11* mRNA levels by real-time qPCR in anti-miR19b–transduced NSCLC cells. Results are presented as mean fold change relative to the control ±SD, normalized to GAPDH (n = 3). **c** Western blot analysis of pre-miR-19b and anti-miR19b–transduced NSCLC cells using PPP2R5E and BCL2L11 specific antibodies. Protein levels were normalized to α-tubulin and GAPDH and presented relative to the control. **d** PP2A activity assay of pre-miR19b- and anti-miR19b–transduced cells relative to the control (n = 3). *, p < 0.05; **, p < 0.01; ***, p < 0.001

Gene network analysis based on the regulation profile of the input elements from the phosphokinase antibody array revealed that serine/threonine phosphatase PP2A, tyrosine phosphatases *PTPN* and *PTEN* are likely to be up-regulated in miR-19b-depleted cells (Additional file 5: Figure S4d). These data are in agreement with the observation that *PTPN*, *PTEN* and PP2A subunits are confirmed or predicted targets of miR-19b, respectively. Targetscan (www.targetscan.org), miRDB (www.mirdb.org) and DianaLab (http://diana.imis.athena-innovation.gr) databases revealed that PP2A regulatory B subunit *PPP2R5E* harbors two sequence motifs in the 3′-untranslated region that are complimentary to the miR-19b seed sequence (Additional file 1: Table S1). To assess whether *PPP2R5E* is a target of miR-19b, luciferase reporter assays were performed. A luciferase reporter construct containing the upstream sequence motif from the *PPP2R5E* 3’UTR (Additional file 1: Table S1) gave rise to enhanced luciferase activity in PC9 cells transduced with antisense-miR19b-construct relative to the control. Conversely, reduced luciferase activity was obtained in cells overexpressing miR-19b (Fig. 3a). In contrast, luciferase activity was refractory to miR-19b expression using constructs, in which the miR-19b binding site had been mutated (Fig. 3a and Additional file 1: Table S1). These results confirm that *PPP2R5E* is a direct target of miR-19b. Consistent with these results, *PPP2R5E* mRNA levels were significantly increased in three EGFR mutant NSCLC cell lines that were stably transduced with antisense-miR19b-construct (Fig. 3b). Under the same conditions, PPP2R5E protein levels were increased in miR-19b attenuated cells. Conversely, PPP2R5E protein was slightly reduced in miR-19b overexpressing PC9 cells, but not HCC4011 and HCC827 cells (Fig. 3c). In addition, PP2A activity was significantly reduced (62%) in miR-19b-overexpressing cells, while attenuation of miR-19b resulted in enhanced PP2A activity (293%, Fig. 3d). Treatment of cell lysates with LB-100, a specific inhibitor of PP2A, completely abrogated PP2A activity, confirming the specificity of the assay. In conclusion, *PPP2R5E* is a relevant target of miR-19b.

The regulated pathway model described in Additional file 5: Figure S4c suggests that miR-19b may also be implicated in the regulation of apoptosis. Interestingly, target prediction databases revealed that *Bcl2L11*, which encodes the apoptosis regulator BIM, is a potential target of miR-19b (Additional file 1: Table S1). Consistent with this finding, attenuation of miR-19b enhanced luciferase activity, while overexpression of miR-19b resulted in reduced luciferase activity of pmirGLO constructs harboring the predicted miR-19b binding site sequence of Bcl2L11 (Fig. 3a), indicating that *Bcl2L11* is a direct target of miR-19b. Mutating the target site abrogated regulation of luciferase activity by miR-19b. These results were confirmed by RT-qPCR (Fig. 3b) and Western blot analysis (Fig. 3c) in three independent *EGFR* mutant NSCLC cell lines.

### miR-19b controls EGFR-induced cellular processes

**Fig. 4:**
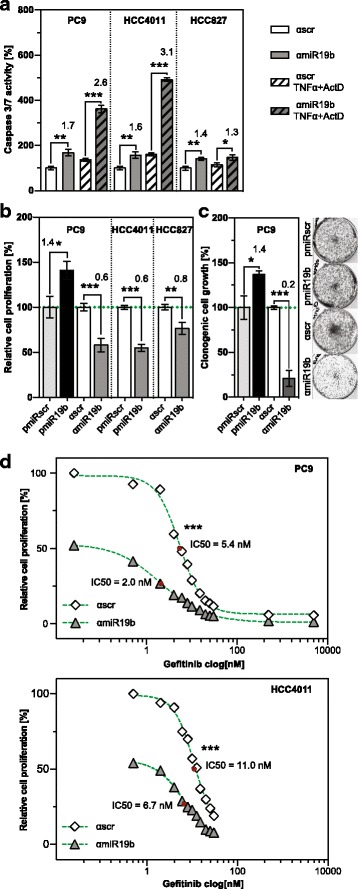
miR-19b regulates EGFR-induced cellular processes. **a** Spontaneous (solid columns) and TNFα/AktD-induced apoptosis (hatched columns) of *EGFR* mutant NSCLC transduced with anti-miR19b construct (n = 3). Apoptosis was analyzed by caspase3/7 cleavage assay 2 h post induction with TNFα/AktD. **b** BrdU incorporation and (**c**) clonogenic growth of pre-miR19b or anti-miR19b–transduced PC9 cells (n = 3). Cells were examined and imaged 8 days post-seeding. *, p < 0.05; **, p < 0.01; ***, p < 0.001, ****, *p* < 0.0001. **d** Clonogenic growth of anti-miR-19b and control-transduced PC9 (upper panel) and HCC4011 cells (bottom panel) at different concentrations of gefitinib. Cell numbers were determined 8 days after incubation with gefitinib. IC50 values were calculated from dose-response curves

To assess the cellular processes regulated by miR-19b, *EGFR* mutant NSCLC cell lines were transduced with anti-miR-19b or miR-19b overexpression constructs. Attenuation of miR-19b levels resulted in enhanced spontaneous apoptosis relative to scrambled control (Fig. 4a, solid white and grey columns and Additional file 6: Figure S5). Tumor necrosis factor alpha (TNFα) in combination with actinomycin D (ActD) elicited an up to 3.1-fold induction of apoptosis in miR-19b-attenuated cells while control-transduced cells were virtually unaffected indicating that endogenous miR-19b levels are sufficient to protect cells from induced apoptosis (Fig. 4a, hatched white and grey columns and Additional file 6: Figure S5).

BrdU incorporation assay was performed to assess proliferation. Pre-miR-19b-transduced cells showed increased BrdU incorporation, while attenuation of miR-19b expression resulted in reduced proliferation in three independent NSCLC cell lines (Fig. 4b). Consistent with this finding, anchorage-dependent clonogenic growth was significantly increased in miR-19b overexpressing cells compared to scrambled control, whereas miR-19b depletion significantly reduced colony formationcapacity (Fig. 4c).

The combinatorial effect of the inhibitors of miR-19b and EGFR, which may either be synergistic, additive, epistatic or suppressive, may provide a deeper insight into the underlying molecular processes [36]. To this end, we assessed clonogenic growth of miR-19b-attenuated cells in the presence of the EGFR inhibitor gefitinib (Fig. 4d and Additional file 7: Figure S6). The half maximal inhibitory concentration (IC50) of gefitinib in control-transduced and anti-miR19b–transduced PC9 cells were 5.4 nM and 2.0 nM, respectively. Thus, attenuation of miR-19b results in a shift in gefitinib sensitivity by a factor of 2.7. Likewise, attenuation of miR-19b in HCC4011 cells gave rise to a shift in the IC50 of gefitinib from 11.0 nM to 6.8 nM. Based on these synergistic effects, we may conclude that EGFR and miR-19b act in the same pathway.

**Fig. 5:**
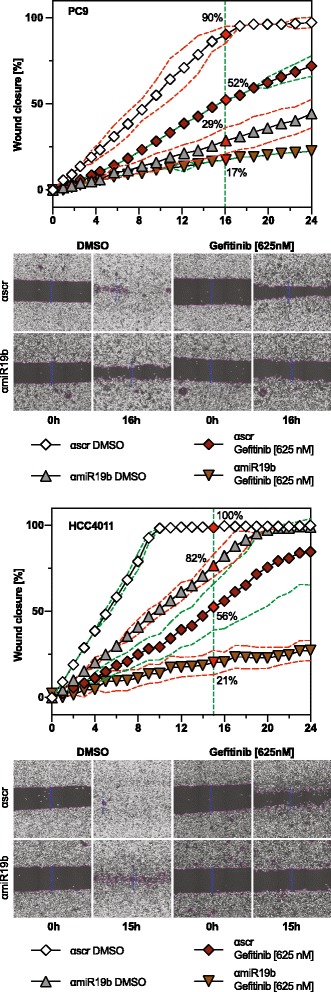
EGFR and miR-19b act together to control cell migration. Wound closure assay of (**a**) PC9 and (**b**) HCC4011 cells. Wound closure was monitored over a period of 24 h in the presence or absence of 0.625 μM gefitinib using the imaging system Cell-IQ. Gefitinib was added immediately after injury of the monolayer. Results are presented as mean ± SD (*n* = 6)

EGFR is also implicated in the regulation of cell migration. Wound closure over time was significantly reduced in anti-miR19b–transduced cells compared to scrambled control (Fig. 5a, b and Additional file 8: Video S1), which is most clearly detected 16 h after wound formation. In both cells lines, gefitinib treatment also resulted in reduced migration, but the combinatorial treatment with anti-miR-19b and gefitinib resulted in a significantly lower migration rate than gefitinib or anti-miR-19b alone. In HCC4011 cells, the combined effect was clearly synergistic (Fig. 5b) while it was additive in the case of PC9 (Fig. 5a). Thus, EGFR and miR-19b act together to control migration.

### miR-19b induces proliferation and apoptosis resistance by targeting PPP2R5E and BCL2L11

**Fig. 6:**
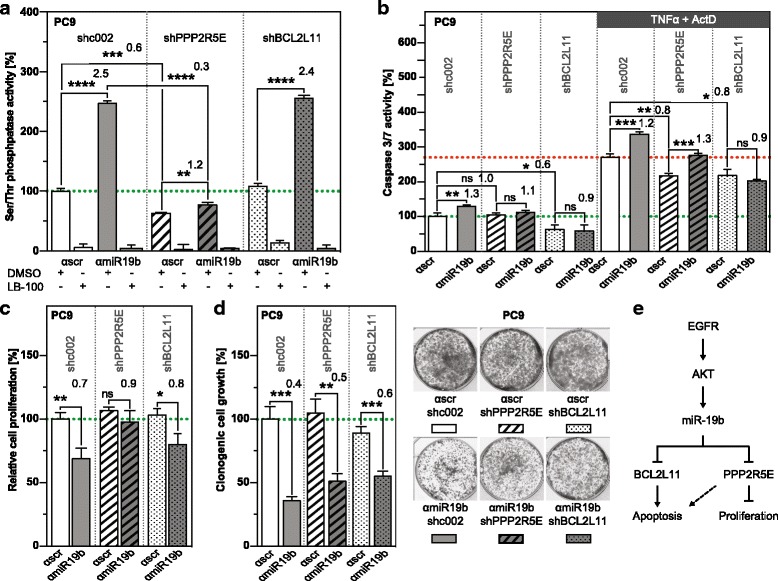
Distinct roles of *PPP2R5E* and *BCL2L11* in PP2A activity, proliferation and apoptosis of miR-19b-attenuated PC9 cells. **a** PP2A activity, (**b**) spontaneous (left panel) and TNFα/ActD-induced (right panel) apoptosis, (**c**) proliferation and (**d**) clonogenic growth of PC9 cells transduced with anti-miR-19b (grey columns) or anti-miR control (αscr, white columns) in combination with *shcontrol* (sh002, solid), *shPPP2R5E* (hatched) and *shBCL2L11* (dotted) (n = 3). Analysis was performed as described in the legend to Fig. 4. **e** Model showing EGFR/AKT-induced miR-19b, its targets and cellular processes in which it is involved. ns, not significant. *, p < 0.05; **, p < 0.01; ***, p < 0.001, ****, p < 0.0001

To assess if *PPP2R5E* and *BCL2L11* are relevant targets of miR-19b, we transduced PC9 cells with anti-miR19b or anti-scrambled control, in combination with *shPPP2R5E*, *shBCL2L11* or *shcontrol* (shc002). In a first experiment, PP2A activity was assessed (Fig. 6a). Attenuation of miR-19b expression resulted in 2.5-fold higher PP2A activity in cells co-transduced with anti-miR-19b and shc002 (solid columns), but a significantly weaker elevation of PP2A activity was observed in cells co-transduced with anti-miR-19b and *shPPP2R5E* (hatched columns). This indicates that miR-19b affects PP2A activity by targeting *PPP2R5E*. PP2A catalytic subunit can form complexes with multiple subunit B isoforms, each of which contributes to PP2A activity. In miR-19b-attenuated cells, PPP2R5E seems to be the major component, since knocking down *PPP2R5E* resulted in 70% less activity (Fig. 6a, compare solid grey and hatched grey columns). In contrast, only ~30% PP2A complexes seem to be associated with PPP2R5E in control-transduced PC9 cells (compare solid white and hatched white columns). As expected, PP2A activity was not altered in the *BCL2L11* knockdown (dotted columns). The specificity of the assay was confirmed by treating cell lysates with LB-100.

PP2A and BIM are both important regulators of apoptosis [37, 38]. In agreement with these findings, knocking down *PPP2R5E* or *BCL2L11* with shRNAs resulted in lower TNFα/ActD-induced apoptosis (Fig. 6b, right panel, compare solid and hatched white columns, or solid and dotted white columns, respectively). In contrast, spontaneous apoptosis was only affected in the *BCL2L11* knockdown (Fig. 6b, left panel, compare solid and dotted white columns). TNFα/ActD-induced apoptosis was increased 1.2 to 1.3-fold relative to control, irrespective of whether cells were co-transduced with anti-miR-19b and shc002 (Fig. 6b, right panel, compare solid white and grey columns) or *shPPP2R5E* and anti-miR-19b (Fig. 6b, right panel, compare hatched columns). In contrast, apoptosis enhancement was abrogated in cells co-transduced with anti-miR-19b and *shBCL2L11* (Fig. 6b, left and right panel, dotted columns). Thus, miR-19b controls apoptosis by targeting *BCL2L11*, whereas *PPP2R5E* seems to be less important (summarized in Fig. 6e).

Conversely, miR-19b-induced proliferation was mainly due to targeting *PPP2R5E* (Fig. 6c, e). This is based on the finding that reduced BrdU incorporation in anti-miR-19b/shc002 co-transduced cells (Fig. 6c, solid white and grey columns) was reversed in anti-miR-19b/*shPPP2R5E*–co-transduced cells (Fig. 6c, hatched columns). In contrast, knocking down *shBCL2L11* had no effect on miR-19b-regulated proliferation (Fig. 6c, dotted columns).

Enhanced proliferation and reduced apoptosis both contribute to clonogenic growth. Consistent with the results shown in Fig. 4c, attenuation of miR-19b repressed clonogenic growth (Fig. 6d, solid columns), but knocking down *PPP2R5E* or *BCL2L11* only partially reversed clonogenic growth of miR-19b attenuated cells (Fig. 6d, hatched or dotted columns). Thus, PPP2R5E and BCL2L11 act together to control clonogenic growth elicited by miR-19b through regulation of proliferation and apoptosis, respectively.

## Discussion

MiRNAs participate in signaling pathways as signal amplifiers or attenuators and regulate the activity of downstream effector pathways, and allow crosstalk between these pathways (reviewed by [15]). We show by microarrays and bioinformatics analysis that miRNAs that are regulated by the PI3K branch of the EGFR signaling pathway are also effectors of this pathway. In agreement with this finding, miR-100 [39], miR-125b [25, 40] and miR-9 [41], which are induced by the PI3K branch of EGFR, are able to enhance NF-κB activity by targeting TRAF-7, TNFAIP3 and FoxO1, respectively. Likewise, miR-205 induces parallel signaling pathways by enhancing the expression of ERBB3 [42]. The oncomiR-1 cluster, which includes miR-18a, miR-19a, miR-19b, miR-20a and miR-20b, is another prominent example of miRNAs involved in oncogenic processes in different cancer systems. Conversely, miR-181a, which is negatively correlated with PI3K activity, interferes with such processes by targeting oncogenic *KRAS* [43] and *Bcl2* [44]. Thus, PI3K-regulated miRNAs act as downstream effectors of EGFR signaling. Interestingly, pathway analysis of the phosphoproteome dataset of miR-19b-attenuated cells and pathway analysis of the gene target dataset of the top 17 miRNAs that are dysregulated by the PI3K inhibitor revealed very similar biological function diagrams (Additional file 5: Figure S4c). This may suggest that the phenotype elicited by the combination of all PI3K-regulated miRNAs may be recapitulated by the phenotype elicited by miR-19b alone. In conclusion, our results are consistent with a model that PI3K-regulated miRNAs act in a concerted manner to modulate the activity of the EGFR signaling pathway.

Our results indicate that miR-19b and EGFR act together to control proliferation, migration and apoptosis of *EGFR* mutant NSCLC in a synergistic manner, forming part of the same signaling pathway. This was confirmed by Western blot analysis showing enhanced phosphorylation of the effectors of EGFR including ERK, STAT and AKT by miR-19b overexpression. Thus, although miR-19b is induced by the PI3K/AKT branch, it activates all three major branches of EGFR indicating that one role of miR-19b is to link these signaling pathways.

How is this achieved? Phosphoproteomic analysis of miR-19b-attenuated cells pinpoints PP2A as a common regulator of ERK, STAT and AKT signaling by miR-19b. PPP2R5E regulation by miR-19b was confirmed by luciferase reporter assays, RT-qPCR, Western blot analysis and PP2A phosphatase activity assays. Thus, PPP2R5E serves as a hub for miR-19b-mediated crosstalk between these pathways.

PPP2R5E is implicated in enhanced proliferation elicited by miR-19b evident from the observation that enhanced proliferation of NSCLC cells elicited by miR-19b was completely restored in the *PPP2R5E* knockdown. In contrast, targeting *PPP2R5E* proved to be dispensable for apoptosis resistance induced by miR-19b. Consistent with these findings, PPP2R5E inhibits proliferation by dephosphorylation of ERK rather than apoptosis [9, 45]. Interestingly, the proapoptotic BH3-only protein BIM (encoded by *BCL2L11*), which is a master regulator of cell death in cancer cells [38], is a relevant target of miR-19b in spontaneous and TNFα/ActD-induced apoptosis. Enhanced apoptosis in miR-19b-attenuated cells is restored in the *BCL2L11* knockdown. In contrast, clonogenic growth is only partially restored by targeting either *PPP2R5E* or *BCL2L11*. One explanation for this finding may be that clonogenic growth is affected by both proliferation and apoptosis, and that only one of both processes is restored in a single *PPP2R5E* or *BCL2L11* knockdown. *PTEN*, a well-established target of miR-19b [46], may potentially corroborate with PPP2R5E and BCL2L11 in miR-19b-induced processes. It remains to be shown if enhanced migration elicited by miR-19b is due to targeting *PTEN* [21], *PPP2R5E* (our study) or a combination of both.

Enforced expression of miR-19b triggers epithelial-mesenchymal transition (EMT) [21]. However, in contrast to our findings and findings obtained by others [20], Li et al. reported that miR-19b overexpression was also responsible for reduced proliferation of the NSCLC cell line A549 [21]. This could be due to off-target effects upon high level expression of miR-19b or cell-type-specific effects. Alternatively, EMT and reduced proliferation may appear during a later period following miR-19b induction. We found that miR-19b overexpressing cells lost their proliferation phenotype upon long term culture, but this was not associated with the appearance of EMT markers (data not shown).

Novel forms of therapies aiming at reactivating PP2A may become important for the treatment of lung cancer in the future. Activators of PP2A such as SMAPs (reviewed by [47]) or inhibitors of negative regulators such as bortezomib or erlotinib, that restore PP2A activity by targeting CIP2A [48], are currently tested in clinical phase I/II studies. These drugs could possibly be exploited for the therapy of EGFR or KRAS-driven NSCLC. One potential drawback may be that all PP2A holoenzymes are equally affected using these pharmacological approaches which may also have an impact on normal tissue. We found that PPP2R5E contributed to 30% PP2A activity in PC9 cells, but PP2A activity was significantly enhanced in miR-19b-attenuated cells which was associated with reduced clonogenic growth. In addition, we found that attenuation of miR-19b sensitized cells to gefitinib treatment. Thus, administering antagomiRs to block enhanced levels of miR-19b may be an interesting alternative therapeutic option as it specifically restores *PPP2R5E* expression in the tumour tissue.

## Conclusion

We report that miR-19b acts with other PI3K-regulated miRNAs in a concerted manner as signal amplifiers to modulate the activity of the EGFR pathway. Serine/threonine phosphatase PP2A as well as BCL2L11 were defined as targets of miR-19b which serve as hubs allowing cross-talk between signaling pathways. A deeper understanding of underlying molecular processes of EGFR signaling involving miRNAs may provide insights into improving the management of *EGFR*-mutant lung cancer patients treated with TKIs. In addition, this work may have therapeutic implications since targeting miR-19b may be a means of affecting PP2A expression and thereby modulating the activity of EGFR signaling.

## Additional files

Additional file 1: **Table S1.** TargetScanHuman Prediction of microRNA hsa-miR-19b-3p targets. (PDF 38 kb)

Additional file 2: **Figure S1.** Pathway analysis of PI3K-regulated microRNAs. a Top 24 regulated pathways sorted by their significance. The greyscale represents the degree of regulation. *P*-values are shown as reverse log format. Ratios reflect the number of the regulated elements, which are predicted as a target, divided by the total number of pathway elements. The –log10 *p*-value cut off was set to 1.3. **b** Word cloud representing pathway elements (mRNA). The font size of a pathway element is characterized by its frequency of occurrence in the pathway analysis data set. The colors are not corresponding to any feature. Pathways elements are ordered alphabetically from left to right. **c** Representation of biological functions corresponding to pathways built based on miRNA targets. Number of pathways participating in each biological function is indicated on the axis of the radar graph. (PDF 4333 kb)

Additional file 3: **Figure S2.** Cell viability of NSCLC cells in the presence of different TKIs. Cell viability was assessed using metabolic resazurin assay (Sigma-Aldrich). In brief cells were seeded at a density of 1500 cells/well in 96-well plate. Treatment was started 24 h post seeding, with inhibitors at the given concentrations shown in the figure over a time period of 72 h. Cell viability was assayed 3 h after adding resazurin using an infinite 200 reader (Tecan, Maennedorf, Switzerland). Results are presented as mean relative viability relative to DMSO treated cells ±SD, (*n* = 3). (PDF 594 kb)

Additional file 4: **Figure S3.** miR-19b expression levels of pre-miR-19b and antimiR-19b-transduced NSCLC cells. miR-19b expression levels were analyzed by real-time qPCR relative to RNU48. Results are presented as mean ± SD (n = 3). *, *p* < 0.05; **, *p* < 0.01; ***, *p* < 0.001. (PDF 151 kb)

Additional file 5: **Figure S4.** Pathway analysis based on the dataset from the phosphatase array of miR-19b attenuated PC9 cells. a Top 25 regulated pathways sorted by their significance. Color intensity represents the degree of regulation. Orange color represents activation and blue color represents inhibition of a pathway, white columns indicates minimum regulatory direction. b Relationship between the main upstream regulator EGF and the downstream effectors based on phospho-array data. Elements that are inhibited by de-phosphorylation are indicated in green and elements that are activated by de-phosphorylation are indicated in orange. **c** Comparison of regulated biological functions based on phosphatase array dataset (red) with microRNA expression dataset (green). Number of pathways involved in each biological function is indicated on the axis of the radar graph. The overlap of the two graphs is shown in brown. **d** Gene network analysis of upregulated phosphatases (orange symbols), which serve as hubs for responsive elements such as kinases (green symbols). Inhibitory interactions are indicated by blue lines and activating interactions are indicated by orange lines *p* < 0.05. (PDF 2995 kb)

Additional file 6: **Figure S5.** miR-19b confers apoptosis resistance in EGFR mutant NSCLC **cells. a** Representative examples of dot plots and densitometric plots of PC9 cells transduced with GFP/anti-miR-19b (αmiR19b) or GFP/anti-miR scramble control (αscr) in the presence (TNFα+ActD) or absence (cRPMI) of an apoptotic trigger. GFP-positive cells were analysed for Annexin V / propidium iodide (Annexin V / PI) by flow cytometry. **b** Percent Annexin V-positive, PI-positive and AnnexinV/PI-negative cells of three EGFR mutant NSCLC cell lines in the presence or absence of an apoptotic trigger (n = 3). (PDF 621 kb)

Additional file 7: **Figure S6.** Clonogenic growth in the presence of gefitinib. Images of 6-well plates 8 days post seeding captured from the clonogenic growth assay described in Fig. 4D. Experiments performed with PC9 cells are shown in the upper part and experiments performed with HCC4011 cells are shown in the bottom of the figure. Gefitinib concentrations are indicated below the images. (PDF 5244 kb)

Additional file 8: **Video S1.** Migration is reduced in miR-19b attenuated cells. Wound healing was analysed by kinetic live cell imaging using a cell-IQ instrument. Video 1A showing anti-miR-transduced. PC9 cells (green) and control (red) and video 1B showing anti-miR scramble control transduced PC9 cells (green) and control (red) are appended to the supplemental material. (ZIP 6645 kb)

## Abbreviations

BrdU: 5-bromo-2-deoxyuridine
EGFR: Epithelial growth factor receptor
IPA: Ingenuity Pathway Analysis
miRNA: microRNA
NSCLC: Non-small-cell lung carcinoma
TKI: Tyrosine kinase inhibitor
