# Supplementary material for: miR-19b enhances proliferation and apoptosis resistance via the EGFR signaling pathway by targeting PP2A and BIM in non-small cell lung cancer
Source: Mol Cancer. 2018 Feb 19;17:44. doi: 10.1186/s12943-018-0781-5 (PMC5817797; doi:10.1186/s12943-018-0781-5)
Supplement: Supplementary file 1 — Table S1. TargetScanHuman Prediction of microRNA hsa-miR-19b-3p targets. (PDF 38 kb) [file 12943_2018_781_MOESM1_ESM.pdf]

Suppl. TableS1: TargetScanHuman Prediction of microRNA hsa-miR-19b-3p targets.

**PPP2R5E (ENST00000337537.7)**	Predicted consequential pairing of target region (top) and miRNA (bottom)	Site type	Context++ score	Context++ score percentile	Weighted context++ score	Conserved branch length	P_{CT}
Position 505-512 of PPP2R5E 3'UTR<br>has-miR-19b-3p	5' ...UUAACAUCUUUUUCUUUUGCACA...<br>||||||| 3' AGUCAAAACGUAUCUAAACGUGU	8mer	-0.34	96	-0.34	4.765	0.84
Position 3584-3591 of PPP2R5E 3'UTR<br>has-miR-19b-3p	5' ...AUCACUGAUGUUUUAUUUGCACA...<br>||||||| 3' AGUCAAAACGUAUCUAAACGUGU	8mer	-0.21	86	-0.04	3.108	0.49
**Luciferase target construct**	Predicted target site (top) and mutated target site cloned into luciferase reporter construct (bottom)						
Luc PPP2R5E TS<br>Luc PPP2R5E mTS	5'-TCGAGTTTAACATCTTTTTCTTTTGCACATCTTCCTGAGTTGAATGTCCT-3'<br>********<br>5'-TCGAGTTTAACATCTTTTTCGGGGTACACTCTTCCTGAGTTGAATGTCCT-3'						
**BCL2L11 (ENTST00000393256.7)**	Predicted consequential pairing of target region (top) and miRNA (bottom)	Site type	Context++ score	Context++ score percentile	Weighted context++ score	Conserved branch length	P_{CT}
Position 4092-4098 of BCL2L11 3'UTR<br>has-miR-19b-3p	5' ...CUGGCUUACUUGUGUUUUGCACA...<br>||||||| 3' AGUCAAAACGUAUCUAAACGUGU	7mer m8	-0.23	88	-0.06	7.317	0.83
**Luciferase target construct**	Predicted target site (top) and mutated target site cloned into luciferase reporter construct (bottom)						
Luc BCL2L11 TS<br>Luc BCL2L11 mTS	5'-TCGAGGTTTCCCTGGCTTACTTGTGTTTTGCACTGATGAATTTTGACT-3'<br>*********<br>5'-TCGAGGTTTCCCTGGCTTACTTGTTGGGGTACATGATGAATTTTGACT-3'						

Context++ score and features that contribute to the context++ score are evaluated as in (Agarwal et al., 2015)
Conserved branch lengths and PTC are evaluated as in (Friedman et al., 2009), with an expanded 84-species alignment as described in (Agarwal et al., 2015).
Table was adapted from targetscan.org (May 22nd 2017).
