# Supplementary material for: miR-19b enhances proliferation and apoptosis resistance via the EGFR signaling pathway by targeting PP2A and BIM in non-small cell lung cancer
Source: Mol Cancer. 2018 Feb 19;17:44. doi: 10.1186/s12943-018-0781-5 (PMC5817797; doi:10.1186/s12943-018-0781-5)
Supplement: Supplementary file 2 — Figure S1. Pathway analysis of PI3K-regulated microRNAs. a Top 24 regulated pathways sorted by their significance. The greyscale represents the degree of regulation. P-values are shown as reverse log format. Ratios reflect the number of the regulated elements, which are predicted as a target, divided by the total number of pathway elements. The –log10 p-value cut off was set to 1.3. b Word cloud representing pathway elements (mRNA). The font size of a pathway element is characterized by its frequency of occurrence in the pathway analysis data set. The colors are not corresponding to any feature. Pathways elements are ordered alphabetically from left to right. c Representation of biological functions corresponding to pathways built based on miRNA targets. Number of pathways participating in each biological function is indicated on the axis of the radar graph. (PDF 4333 kb) [file 12943_2018_781_MOESM2_ESM.pdf]

Suppl. Fig. S1

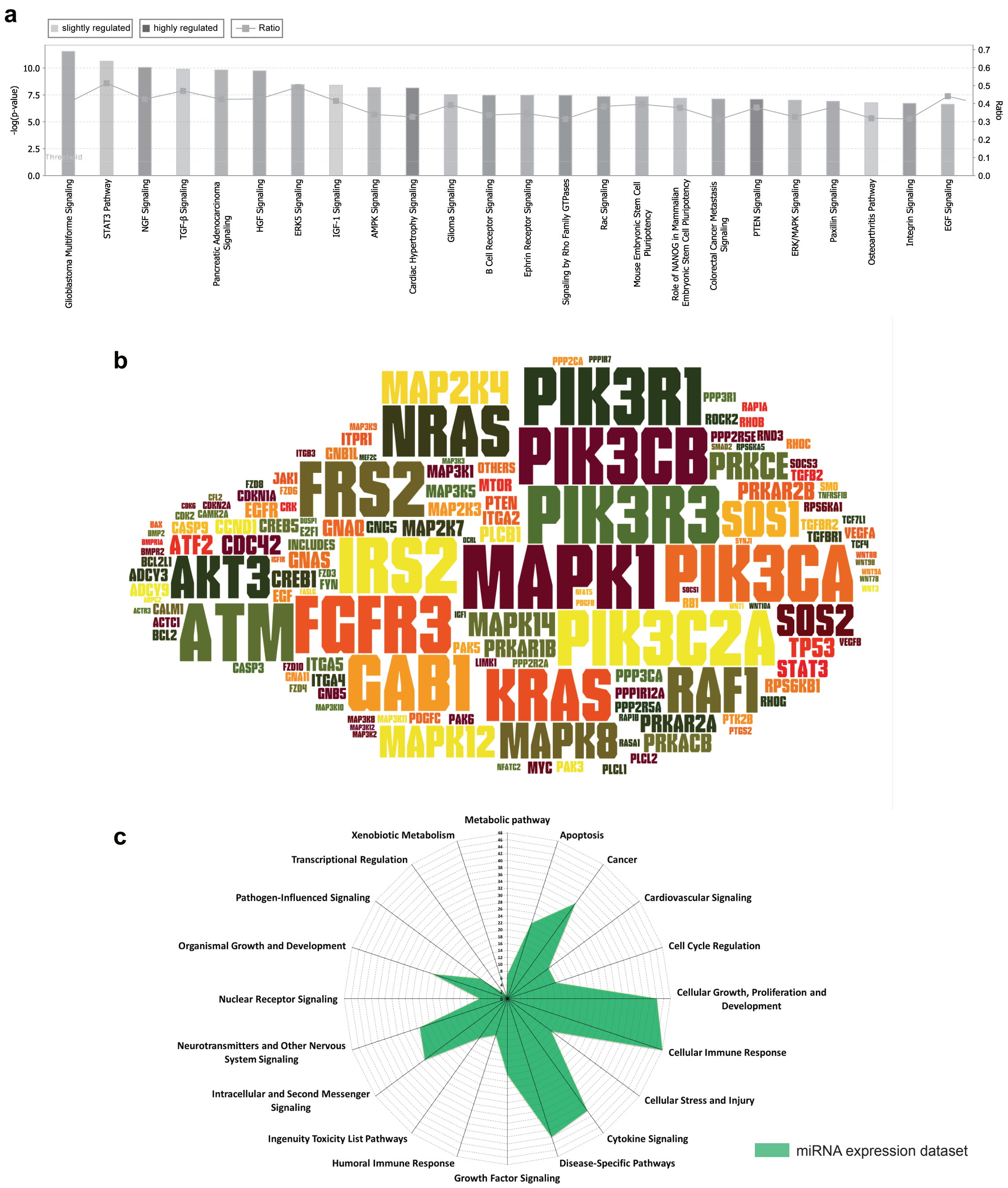

Suppl. Fig. S1: Pathway analysis of PI3K-regulated microRNAs. a Top 25 regulated pathways sorted by their significance. The greyscale represents the degree of regulation. P-values are shown as reverse log format. Ratios reflect the number of the regulated elements, which are predicted as a target, divided by the total number of pathway elements. The –log10 p-value cut off was set to 1.3. **b** Word cloud representing pathway elements (mRNA). The font size of a pathway element is characterized by its frequency of occurrence in the pathway analysis data set. The colors are not corresponding to any feature. Pathways elements are ordered alphabetically from left to right. **c** Representation of biological functions corresponding to pathways built based on miRNA targets. Number of pathways participating in each biological function is indicated on the axis of the radar graph.
