# Supplementary material for: miR-19b enhances proliferation and apoptosis resistance via the EGFR signaling pathway by targeting PP2A and BIM in non-small cell lung cancer
Source: Mol Cancer. 2018 Feb 19;17:44. doi: 10.1186/s12943-018-0781-5 (PMC5817797; doi:10.1186/s12943-018-0781-5)
Supplement: Supplementary file 3 — Figure S2. Cell viability of NSCLC cells in the presence of different TKIs. Cell viability was assessed using metabolic resazurin assay (Sigma-Aldrich). In brief cells were seeded at a density of 1500 cells/well in 96-well plate. Treatment was started 24 h post seeding, with inhibitors at the given concentrations shown in the figure over a time period of 72 h. Cell viability was assayed 3 h after adding resazurin using an infinite 200 reader (Tecan, Maennedorf, Switzerland). Results are presented as mean relative viability relative to DMSO treated cells ±SD, (n = 3). (PDF 594 kb) [file 12943_2018_781_MOESM3_ESM.pdf]

Suppl. Fig. S2

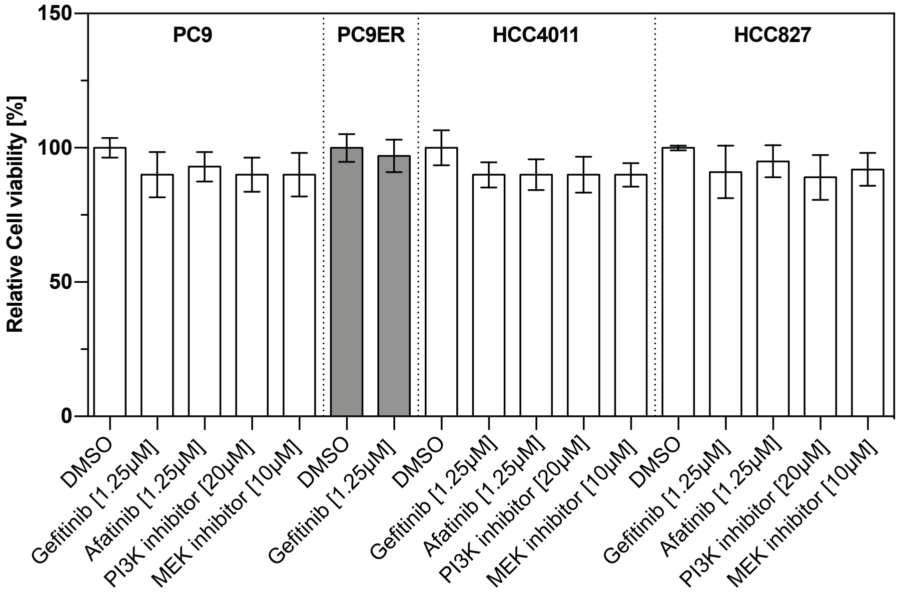

Suppl. Fig. S2: Cell viability of NSCLC cells in the presence of different TKIs. Cell viability was assessed using metabolic resazurin assay (Sigma-Aldrich). In brief cells were seeded at a density of 1500 cells/well in 96-well plate. Treatment was started 24h post seeding, with inhibitors at the given concentrations shown in the figure over a time period of 72h. Cell viability was assayed 3h after adding resazurin using an infinite 200 reader (Tecan, Maennedorf, Switzerland). Results are presented as mean relative viability relative to DMSO treated cells ±SD, (n=3).
