# Supplementary material for: miR-19b enhances proliferation and apoptosis resistance via the EGFR signaling pathway by targeting PP2A and BIM in non-small cell lung cancer
Source: Mol Cancer. 2018 Feb 19;17:44. doi: 10.1186/s12943-018-0781-5 (PMC5817797; doi:10.1186/s12943-018-0781-5)
Supplement: Supplementary file 4 — Figure S3. miR-19b expression levels of pre-miR-19b and antimiR-19b-transduced NSCLC cells. miR-19b expression levels were analyzed by real-time qPCR relative to RNU48. Results are presented as mean ± SD (n = 3). *, p < 0.05; **, p < 0.01; ***, p < 0.001. (PDF 151 kb) [file 12943_2018_781_MOESM4_ESM.pdf]

Suppl. Fig. S3

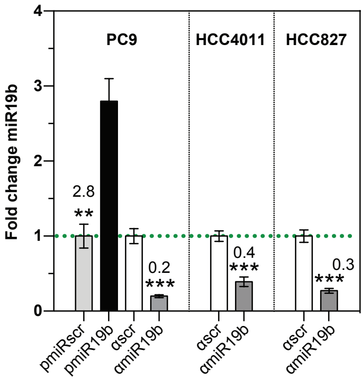

Supplementary Figure S3: miR-19b expression levels of pre-miR-19b and anti-miR-19b-transduced NSCLC cells. miR-19b expression levels were analyzed by real-time qPCR relative to RNU48. Results are presented as mean ±SD (n=3). *, p<0.05; **, p<0.01; ***, p<0.001.
