# Supplementary material for: miR-19b enhances proliferation and apoptosis resistance via the EGFR signaling pathway by targeting PP2A and BIM in non-small cell lung cancer
Source: Mol Cancer. 2018 Feb 19;17:44. doi: 10.1186/s12943-018-0781-5 (PMC5817797; doi:10.1186/s12943-018-0781-5)
Supplement: Supplementary file 5 — Figure S4. Pathway analysis based on the dataset from the phosphatase array of miR-19b attenuated PC9 cells. a Top 25 regulated pathways sorted by their significance. Color intensity represents the degree of regulation. Orange color represents activation and blue color represents inhibition of a pathway, white columns indicates minimum regulatory direction. b Relationship between the main upstream regulator EGF and the downstream effectors based on phospho-array data. Elements that are inhibited by de-phosphorylation are indicated in green and elements that are activated by de-phosphorylation are indicated in orange. c Comparison of regulated biological functions based on phosphatase array dataset (red) with microRNA expression dataset (green). Number of pathways involved in each biological function is indicated on the axis of the radar graph. The overlap of the two graphs is shown in brown. d Gene network analysis of upregulated phosphatases (orange symbols), which serve as hubs for responsive elements such as kinases (green symbols). Inhibitory interactions are indicated by blue lines and activating interactions are indicated by orange lines p < 0.05. (PDF 2995 kb) [file 12943_2018_781_MOESM5_ESM.pdf]

Suppl. Fig. S4

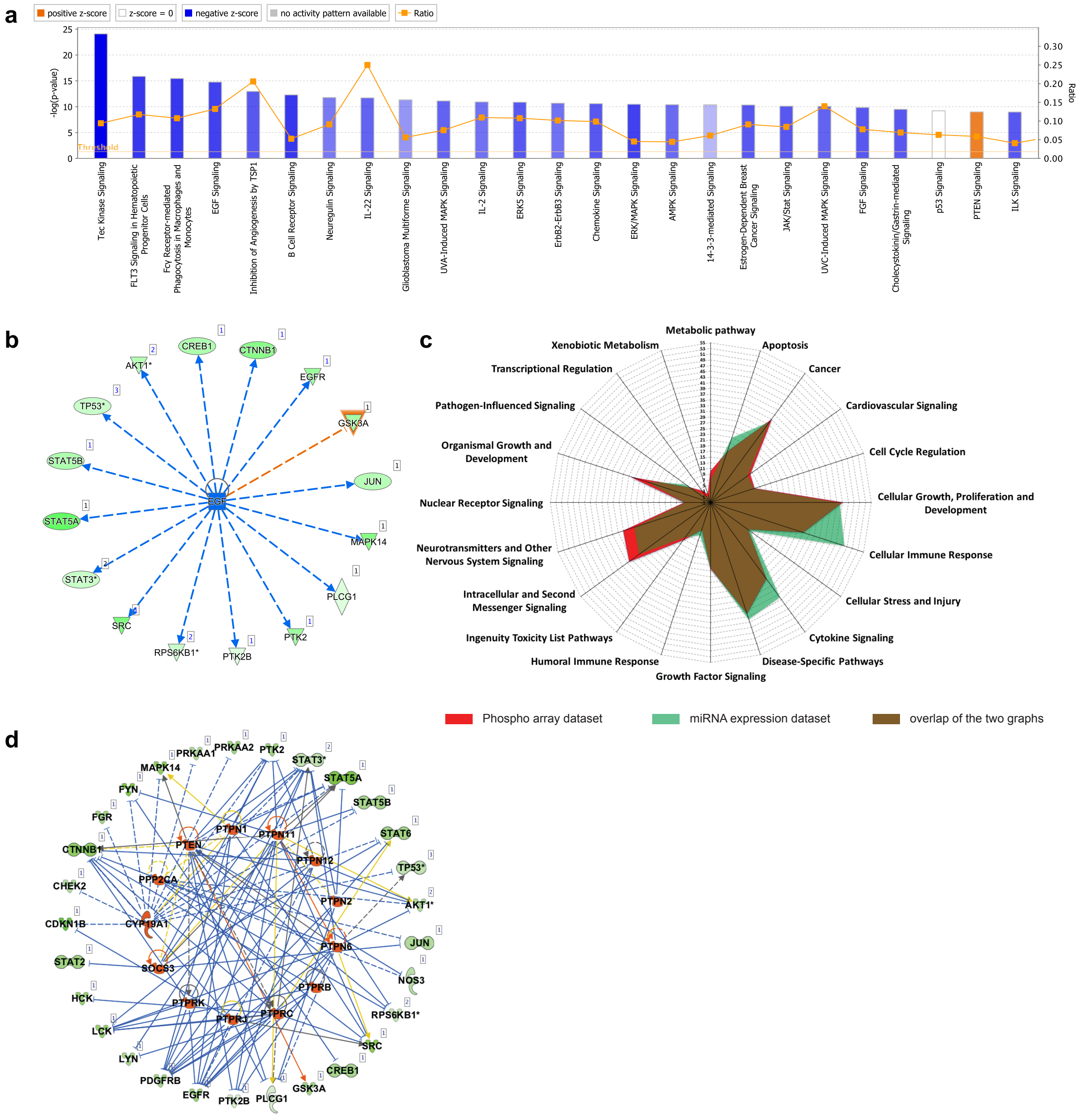

Suppl. Fig. S4: Pathway analysis based on the dataset from the phosphatase array of miR-19b attenuated PC9 cells. a Top 25 regulated pathways sorted by their significance. Color intensity represents the degree of regulation. Orange color represents activation and blue color represents inhibition of a pathway, white columns indicates minimum regulatory direction. **b** Relationship between the main upstream regulator EGF and the downstream effectors based on phospho-array data. Elements that are inhibited by de-phosphorylation are indicated in green and elements that are activated by de-phosphorylation are indicated in orange. **c** Comparison of regulated biological functions based on phosphatase array dataset (red) with microRNA expression dataset (green). Number of pathways involved in each biological function is indicated on the axis of the radar graph. The overlap of the two graphs is shown in brown. **d** Gene network analysis of upregulated phosphatases (orange symbols), which serve as hubs for responsive elements such as kinases (green symbols). Inhibitory interactions are indicated by blue lines and activating interactions are indicated by orange lines p<0.05.
