# Supplementary material for: miR-19b enhances proliferation and apoptosis resistance via the EGFR signaling pathway by targeting PP2A and BIM in non-small cell lung cancer
Source: Mol Cancer. 2018 Feb 19;17:44. doi: 10.1186/s12943-018-0781-5 (PMC5817797; doi:10.1186/s12943-018-0781-5)
Supplement: Supplementary file 6 — Figure S5. miR-19b confers apoptosis resistance in EGFR mutant NSCLC cells. a Representative examples of dot plots and densitometric plots of PC9 cells transduced with GFP/anti-miR-19b (αmiR19b) or GFP/anti-miR scramble control (αscr) in the presence (TNFα+ActD) or absence (cRPMI) of an apoptotic trigger. GFP-positive cells were analysed for Annexin V / propidium iodide (Annexin V / PI) by flow cytometry. b Percent Annexin V-positive, PI-positive and AnnexinV/PI-negative cells of three EGFR mutant NSCLC cell lines in the presence or absence of an apoptotic trigger (n = 3). (PDF 621 kb) [file 12943_2018_781_MOESM6_ESM.pdf]

Suppl. Fig. S5

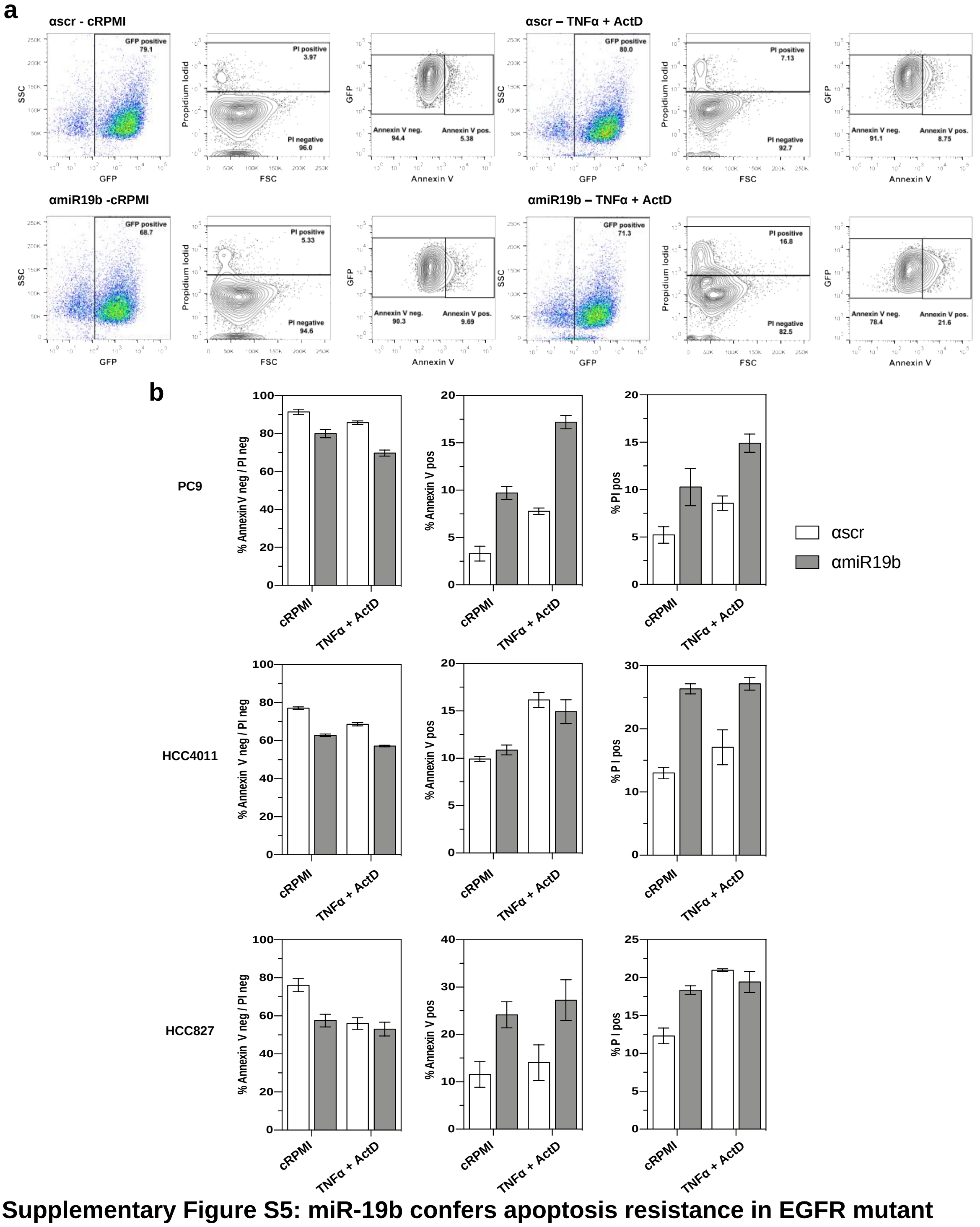

Supplementary Figure S5: miR-19b confers apoptosis resistance in EGFR mutant NSCLC cells. a Representative examples of dot plots and densitometric plots of PC9 cells transduced with GFP/anti-miR-19b (αmiR19b) or GFP/anti-miR scramble control (αscr) in the presence (TNFα+ActD) or absence (cRPMI) of an apoptotic trigger. GFP-positive cells were analysed for Annexin V / propidium iodide (Annexin V / PI) by flow cytometry. **b** Percent Annexin V-positive, PI-positive and AnnexinV/PI-negative cells of three EGFR mutant NSCLC cell lines in the presence or absence of an apoptotic trigger (n=3).
