# Supplementary material for: miR-19b enhances proliferation and apoptosis resistance via the EGFR signaling pathway by targeting PP2A and BIM in non-small cell lung cancer
Source: Mol Cancer. 2018 Feb 19;17:44. doi: 10.1186/s12943-018-0781-5 (PMC5817797; doi:10.1186/s12943-018-0781-5)
Supplement: Supplementary file 7 — Figure S6. Clonogenic growth in the presence of gefitinib. Images of 6-well plates 8 days post seeding captured from the clonogenic growth assay described in Fig. 4D. Experiments performed with PC9 cells are shown in the upper part and experiments performed with HCC4011 cells are shown in the bottom of the figure. Gefitinib concentrations are indicated below the images. (PDF 5244 kb) [file 12943_2018_781_MOESM7_ESM.pdf]

Suppl. Fig. S6

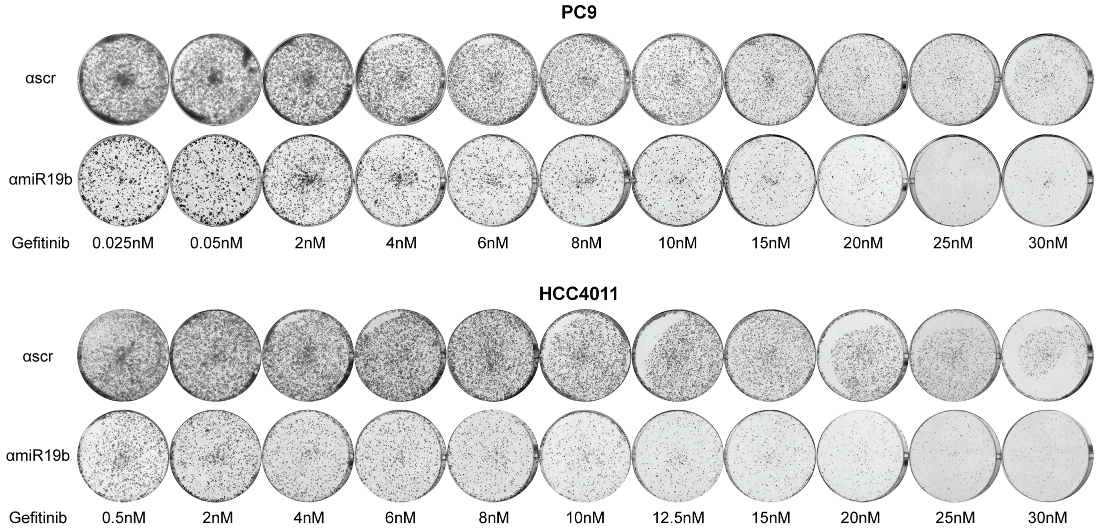

Suppl. Fig. S6: Clonogenic growth in the presence of gefitinib. Images of 6-well plates 8 days post seeding captured from the clonogenic growth assay described in Fig. 4D. Experiments performed with PC9 cells are shown in the upper part and experiments performed with HCC4011 cells are shown in the bottom of the figure. Gefitinib concentrations are indicated below the images.
