# Supplementary material for: miR-19b enhances proliferation and apoptosis resistance via the EGFR signaling pathway by targeting PP2A and BIM in non-small cell lung cancer
Source: Mol Cancer. 2018 Feb 19;17:44. doi: 10.1186/s12943-018-0781-5 (PMC5817797; doi:10.1186/s12943-018-0781-5)
Supplement: Supplementary file 8 — Video S1. Migration is reduced in miR-19b attenuated cells. Wound healing was analysed by kinetic live cell imaging using a cell-IQ instrument. Video 1A showing anti-miR-transduced. PC9 cells (green) and control (red) and video 1B showing anti-miR scramble control transduced PC9 cells (green) and control (red) are appended to the supplemental material. (ZIP 6645 kb) [file 12943_2018_781_MOESM8_ESM.zip › Additional file 8 Suppl_video 1.pdf]

Suppl. Digital video S1

Migration is reduced in miR-19b attenuated cells. Wound healing was analysed by kinetic live cell imaging using a cell-IQ instrument. Video 1A showing anti-miR-transduced PC9 cells (green) and control (red) and video 1B showing anti-miR scramble control transduced PC9 cells (green) and control (red) are appended to the supplemental material.
